# A Photoluminescence-Based Field Method for Detection of Traces of Explosives

**DOI:** 10.1100/tsw.2004.126

**Published:** 2004-08-26

**Authors:** E. Roland Menzel, Laird W. Menzel, Jake R. Schwierking

**Affiliations:** Center for Forensic Studies, Physics Department, Texas Tech University, Lubbock, TX 79409-1051, USA

**Keywords:** explosives, color tests, lasers, photoluminescence, fluorescence, ETK^plus^, europium, quantum dots, time-resolved imaging

## Abstract

We report a photoluminescence-based field method for detecting traces of explosives. In its standard version, the method utilizes a commercially available color spot test kit for treating explosive traces on filter paper after swabbing. The colored products are fluorescent under illumination with a laser that operates on three C-size flashlight batteries and delivers light at 532 nm. In the fluorescence detection mode, by visual inspection, the typical sensitivity gain is a factor of 100. The method is applicable to a wide variety of explosives. In its time-resolved version, intended for *in situ* work, explosives are tagged with europium complexes. Instrumentation-wise, the time-resolved detection, again visual, can be accomplished in facile fashion. The europium luminescence excitation utilizes a laser operating at 355 nm. We demonstrate the feasibility of CdSe quantum dot sensitization of europium luminescence for time-resolved purposes. This would allow the use of the above 532 nm laser.

## INTRODUCTION

In a previous article[1], we described the detection of trace quantities of explosives via photoluminescence. Ultimately, this work aimed at a true field method for sensitive trace explosives detection; quick, compact, portable, requiring no external power source[2]. At the time, we did not have on hand the requisite photoluminescence excitation light source. Thus, we mimicked such a source with an argon ion laser (which most certainly is not portable) operated at intensities low enough to be comparable to what might be achieved in a field scenario. The rationale for attempting to detect traces of explosives by photoluminescence is that photoluminescence techniques quite generally are far more sensitive than the corresponding (absorption/reflection-based) colorimetric techniques. An example in the forensic science arena is the sensitivity gain of photoluminescence detection of fingerprints as compared to the traditional colorimetric detection. The current colorimetric true field method for trace explosives detection consists of chemical color spot tests, as epitomized by the $ETK^{plus}$ kit[3,4] used by the Israel

Police and other law enforcement agencies. It is commercially available today in the U.S. through Homeland Security Products, LLC., 307 East Shore Rd., Great Neck, NY 11023, Tel: 516 466-5210. Quite apart from the obvious forensic science and antiterrorism context, such field methodology is of interest also in other areas, e.g., environmental decontamination[5]. In our earlier work, we utilized the ETK kit as a starting point. The products formed with this kit were found to be fluorescent and, via the fluorescence, a sensitivity gain typically 100-fold over the color sensitivity was achieved for the few explosives tested[1]. Reasons why the colored ETK products are also fluorescent have been discussed previously[1]. In this paper, we report on the actual field method implementation, using a compact fluorescence excitation laser that operates (in the green) on ordinary flashlight batteries. We report on the results obtained with a more comprehensive number of explosives.

Normally, the field procedure would consist of swabbing the article of interest with filter paper and then spotting the paper with the pertinent reagent. However, one can envision instances in which this procedure is not appropriate, instances where *in situ* detection is needed. Now one has to contend with background color and background fluorescence, which tend to be ubiquitous. In the photoluminescence sense, one would then employ time-resolved detection that utilizes photoluminescence explosives tagging, which results in luminescence lifetime long compared to the background fluorescence lifetime. Time-resolved techniques, in turn, are well known. They have been applied in the forensic science context in fingerprint detection, for instance[6]. Generally, lanthanide complexes are ideal taggants for time-resolved purposes because of their exceptionally long luminescence lifetimes. Europium complexes are particularly effective. Accordingly, we earlier explored explosives tagging with such complexes[1]. The tagging was shown to be feasible. The sensitivity was not impressive, however, because of lack of chemical optimization and because the photoluminescence excitation required UV light, which was of very low intensity in the hand-held UV lamp then employed. That lamp, moreover, is not useful for time-resolved work. In this paper, we report on the resolution of the sensitivity problem, and on the time-resolved implementation also via replacement of the UV lamp with a laser. We report as well on the feasibility of sensitization of europium photoluminescence amenable to the very same green light source that is used in our standard field procedure discussed in connection with the ETK kit. This is desirable in the long run. A simple device for visual time-resolved observation that in principle lends itself to incorporation into our standard field device to add the time-resolved capability, as needed, was described a long time ago in connection with fingerprint detection[7] for small samples. A fairly straightforward mechanical modification[8] suffices to handle large articles or surfaces, as required for *in situ* explosives trace detection.

## ETK APPROACH

The literature that accompanies the ETK color spot test kit distinguishes between four types of explosive. Explosives of Type I, which include, among others, dinitrotoluene (DNT), trinitrotoluene (TNT), and tetryl, are probed by the reagent of Tube 1 of the kit. If no color is developed on spotting with this reagent, then spotting on the same sample with Tube 2 follows. This is of special interest in the present context in terms of fluorescence. The Tube 2 spotting involves alkaline hydrolysis producing nitrite ions that are then detected by the well-known Griess reaction and probes Type II explosives, such as nitroglycerin, nitrocellulose, PETN, RDX, HMX, and SEMTEX. We explicitly mention the Tube 2 chemistry here because, as we shall see, it impacts in the photoluminescence sense Type I and Type IV explosives also. If still no color develops, then spotting, still on the same sample, by Tube 3 follows to probe Type III explosives, which involve inorganic nitrates. If this fails, then a new sample is spotted with Tube 4 to probe Type IV explosives, which involve inorganic chlorates or bromates.

## Materials

We have examined 2,4 DNT and TNT (Type I), nitroglycerin, nitrocellulose, RDX, HMX (Type II), potassium and ammonium nitrate (Type III), and potassium chlorate (Type IV). The 2,4 DNT was obtained from Aldrich (a well-known chemical company). Nitroglycerin tablets, prescribed for certain heart diseases, served as the source of nitroglycerin. Potassium chlorate and nitrate as well as ammonium nitrate, the target ingredient in ANFO (a mixture of ammonium nitrate and fuel oil), were purchased from Aldrich. Single-base smokeless powder, SR 4756, with nitrocellulose as the target ingredient, was purchased from a local gun shop, as was black powder (potassium nitrate being the target ingredient). Samples of the remaining explosives were obtained from the Lubbock County Bomb Squad. This included C4, which is a mixture of RDX and fuel oil.

## Procedure

In order to perform sensitivity studies, samples of the target materials were extracted with acetone. This simply involved pouring the powders into the solvent and stirring for a few minutes in a sonicator. This was done for all samples excepting the nitates and chlorate, which are poorly soluble in acetone. Thus, the extraction for those samples employed water. In some instances, considerable residue remained precipitated at the bottom of the sample flasks after the sonication (filler material). The initial concentrations were 0.5 g of powder in 50 ml of solvent. After sonication, a portion of the supernatant liquid was pipetted and then diluted by successive factors of ten. One drop of each such dilution was then spotted from a capillary tube on Whatman 1 filter paper (4.25 cm dia.) and subsequently reagent-spotted as prescribed by the ETK procedure. Each time, a bare filter paper was spotted at the same time with the ETK reagents in the same manner to serve as control. Whereas it was easy to apply a single drop of sample to the filter paper, we had some difficulty in applying single drops of ETK reagents. It is at times necessary to apply pressure to the ETK tubes, and often more than one drop of reagent hits the filter paper, or the drops vary in size. Moreover, the spreading of liquids on the filter paper varies some, especially when the filter paper is not entirely flat. Thus, in colorimetric and fluorescence detection, one tends to obtain irregularly shaped spots. On repeated experimentation, we found no significant effect on results irrespective of just how much reagent was applied or how big the reacted spot was (within reason). The photoluminescence field device utilizes a compact light-tight box to which the laser is attached[8]. The box has a viewing port equipped with the appropriate filter that blocks reflected laser light and transmits the fluorescence of interest. The box is equipped with a slide for introducing the sample and control filter papers into the chamber for viewing. The size of the box is determined by the minimum distance (roughly 30 cm) at which the eye can focus since, for simplicity, it contains no internal optics (via which the box could be made smaller). In our laboratory experiments, it was usually more efficient to bypass the chamber by turning the whole laboratory into the viewing chamber simply by turning the room lights off and observing the laser-illuminated samples and controls through laser safety goggles equipped with the appropriate (orange) filter. As anticipated already[1], the fluorescence excitation utilizes a frequency-doubled Nd:$YVO_4$ laser operating at 532 nm. This laser (Model 532 GBD, B. E. Meyers & Co., Inc., 14540 NE 91$^{st}$ St., Redmond, WA 98052, www.bemeyers.com) operates on three C-size flashlight batteries. It can be operated in slow pulse, fast pulse, and continuous modes. The latter mode is of interest here. The laser delivers an impressive 130 mW of power in a beam of 1 cm dia. In one set of experiments, the laser beam was expanded to 2 cm dia. with no resulting loss of sensitivity. Once the laser warms up, within a few seconds, the beam profile is essentially circular, with only minor imperfections that for our purposes are inconsequential. Once the batteries wear out, however, the beam profile degrades quite considerably, breaking up into three irregular spots, akin to a circular spot with two fairly wide dark lines through it. Thus, one knows when to replace the batteries. Fig. 1 shows a photo of the laser, with scale in centimeters. Rather than employing the on-off button on the laser, one can operate it with a remote umbilical cord button, which we find convenient. For completeness, Fig. 2 shows this already reported[8] field device.

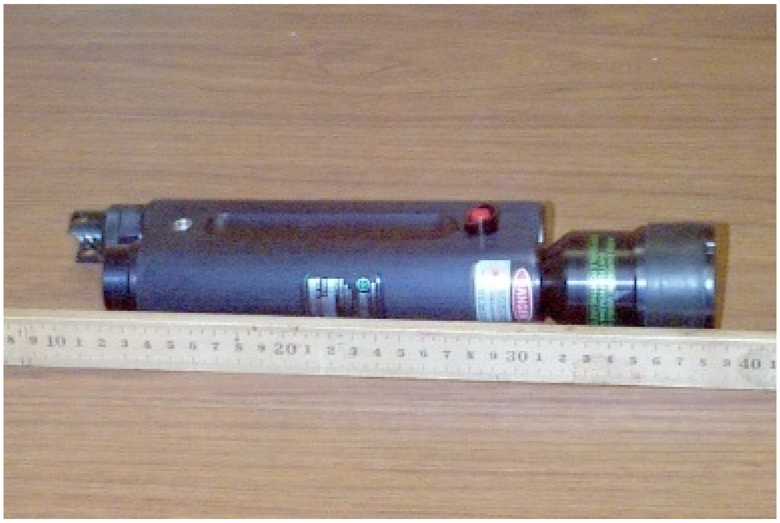

**FIGURE 1**. Photo of battery-operated laser.

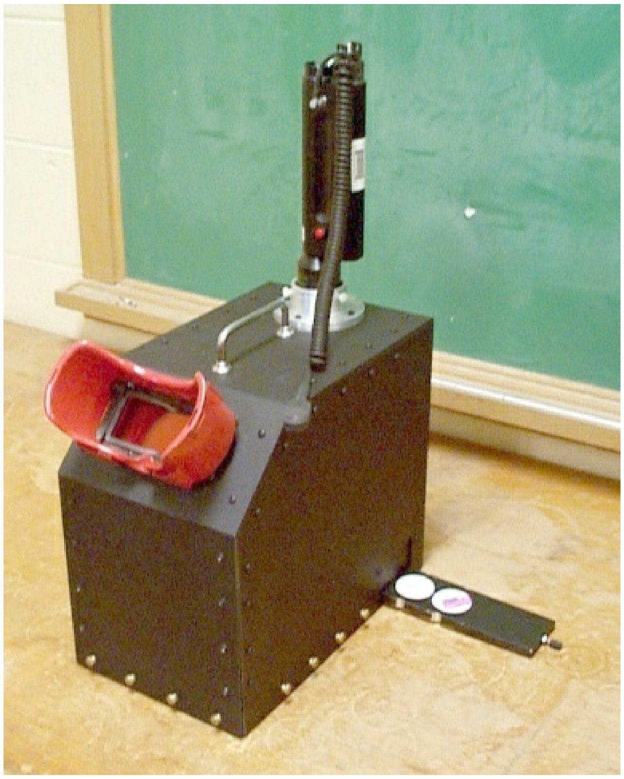

**FIGURE 2**. Field device for explosives photoluminescence detection with ETK.

## RESULTS

We note from the outset that the ETK chemistry is a time-dependent one. It has been noted by us as well as others[9] that color develops in control samples after about 10 min, leading to potential false effects. The main culprit here is Tube 2 of the ETK kit, the Griess reaction, which evidently takes place spontaneously, albeit slowly, whereas in the presence of explosive it is expedited. In the field scenario, this is not serious, in that the field test is intended to be quick. From the photoluminescence perspective, the situation is somewhat worse in that false photoluminescence begins to develop on controls already after about 5 min in any procedure involving ETK Tube 2. Our procedure always involves comparison between sample and control, given that the observed fluorescences invariably appear orange to the eye (through the laser safety filter). Thus, discriminations are based on intensity only. Accordingly, the control photoluminescence development is not so much an issue of a false positive as it is an issue of sensitivity loss. One would thus be tempted to perform successive spottings as quickly as possible. This is detrimental as well, however, in that it helps to let spots settle (by letting solvents evaporate some), lest spots bleed out over large areas of the filter paper to the detriment of color as well as fluorescence observation.

## Type I Explosives

- **2,4 DNT**: Instead of the pink to red colorimetric product indicated in the literature that accompanies the commercial ETK, we obtained a green product. We understand that the formation of this green product is well known. The product changed within a few minutes to grey, and finally after a good many minutes to reddish. No fluorescence was observed during the course of this evolution. The Griess reaction should pertain to Type I explosives. Thus, we followed with Tube 2 ETK spotting. The green product changed to orange on this spotting and this orange product was intensely fluorescent. Time-optimization experimentation showed that Tube 1 spotting should occur about 2 min before Tube 2 spotting, and this became our standard protocol regardless of explosive type (I, II, or III). Once the spotting with Tube 2 was done, fluorescence observation could take place within seconds, certainly within times no more than a minute. Under these conditions, the fluorescence sensitivity gain was three factors of ten in dilution, namely a factor of 1000. The sensitivity assessment simply involved noting the lowest dilution at which color was still noticeable and the lowest dilution at which the observed fluorescence intensity unambiguously exceeded that of the control, processed in the same manner in the same time-span.
- **TNT**: On Tube 1 ETK spotting, this explosive immediately produced a reddish color. No fluorescence was obtained then. On subsequent Tube 2 spotting, in the timed manner described for DNT, orange color developed. Two factors of ten (one hundred times) in sensitivity gain via fluorescence was found.

## Type II Explosives

All Type II explosives behaved the same. Colorimetrically, they yield no color after Tube 1 and pink to red color after Tube 2 according to the ETK literature, to our observation a magenta color. All explosives excepting HMX yielded two factors of ten in sensitivity gain fluorescence-wise. HMX produced three factors of ten sensitivity gain. Both the colorimetric and photoluminescence detection work very nicely with this type of explosive. It is very forgiving to deviations from the reaction time protocols of this article.

## Type III Explosives

The color development after ETK Tube 3 for these nitrate-based explosives was somewhat slow. It was necessary to wait for about 2–3 min after spotting to obtain optimal color (red) and fluorescence results.

For the nitrates themselves, as well as black powder, two factors of ten in sensitivity gain by fluorescence were found.

## *Type IV Explosives*

Here, we only examined potassium chlorate as the active ingredient. As indicated by the ETK literature, blue color developed on spotting with Tube 4. No fluorescence was observed, not surprisingly in that blue and green compounds can hardly be expected to fluoresce under green excitation. However, and though this is not called for by the ETK protocol, we did apply subsequent spotting, after 2 min, with ETK Tube 2. The blue product changed to orange in the center of the spot and to green (mixture of blue and orange) at the periphery of the spot. The spot became fluorescent. One factor of ten in fluorescence sensitivity gain was found.

A brief postreaction ethanol rinse may be applied after Tube 2 spotting. It retards (but does not eliminate) the background fluorescence development, and makes for a crisper observation of the explosive fluorescence, without significant explosive loss. We did not, on this rinsing, find a significant sensitivity gain, however. Postreaction rinses with methanol, acetone, or water were not useful. The matter of postreaction rinsing to inhibit background development altogether is still under investigation.

Table 1 summarizes our results. Quantitative experiments were carried out to determine the absolute sensitivity, in grams of explosive detectable. The one-drop spotting from the capillary tube involved about 1/400 ml. For Types I–III explosives, the limit of the detection was in the 1–0.1 μg/l explosive concentration range. Our detection limit was thus in the $10^{-9}$- to $10^{-10}$-g range for Types I, II, and III explosives. Similarly, it was about $10^{-6}$ g for the Type IV explosive. The corresponding colorimetric sensitivities are in good agreement with what is reported in ref. [4].

**TABLE 1**
**Summary of Explosives Detection Sensitivity Gain by Photoluminescence**

| Expl. type | Explosive | Seensitivity gain (factors of ten) |
|---|---|---|
| I | 2,4 dinitrotoluene<br>trinitrotoluene | 3<br>2 |
| II | RDX<br>HMX<br>C4<br>nitroglycenrin<br>smokeless powder | 2<br>3<br>2<br>2<br>2 |
| III | ANFO<br>black powder | 2<br>2 |
| IV | potassium chlorate | 1 |

Excitation and fluorescence spectra were obtained for several explosives tested. They were quite broad and featureless. The excitation generally peaked at very roughly 550 nm. The fluorescences generally peaked in the 570- to 600-nm range (somewhat concentration dependent). Thus, excitation at 532 nm and visual observation through the standard (orange) laser safety filter for this excitation is a very good overall compromise.

Extraction, dilution, and spotting are fine ways to assess sensitivity, but do not represent the actual field scenario. To place the ETK explosives detection in perspective, we thus placed (as a representative example) a small amount of RDX powder on paper, just barely enough to be visible to the eye. The explosive-containing area of the paper was then successively swabbed with (dry, as opposed to moistened) filter paper and subjected to ETK processing. With successive swabbing, the color development became successively weaker, as to be expected. By the sixth swabbing, no color development at all was found, but on photoluminescence examination, the explosive residue was still quite readily detectable by our field prototype on the sixth swab.

## EUROPIUM APPROACH FOR TIME-RESOLVED DETECTION

### Basic Concepts

As mentioned earlier, *in situ* trace explosives detection in concert with time-resolved visualization has been explored by us via europium ($Eu^{3+}$) complexes. The tagging of the explosive here is ultimately an exercise in coordination chemistry, as discussed previously[1]. If one were to examine the luminescence of common europium compounds, e.g., $EuCl_3.6H_2O$, one would find feeble luminescence only, for two reasons. First, the europium ion absorbs poorly, hence will emit poorly. Second, the water molecules bound to the compound, ubiquitously and strongly so, are notorious luminescence quenchers not only in europium, but in lanthanides generally. However, it has been known since circa WWII that one may chemically bind to europium, and other lanthanides, organic ligands that absorb strongly and then transfer the excitation energy to the lanthanide to produce massively enhanced luminescence. They also tend to exclude water from the formed complex. These ligands are referred to as sensitizing ligands, or donors more generally, and the lanthanide ion, europium in the present instance, is more generally referred to as an acceptor. A critical feature in the donor-acceptor energy transfer is the overlap between the donor fluorescence and the acceptor absorption spectra. Many donors for lanthanide sensitization have been developed over the years, but invariably the donor-acceptor spectral overlap has involved lanthanide acceptor states in the UV spectral region, requiring donors excited with UV light. These acceptor states are not the emitting states of the lanthanides. Rather, cascading from them to the lower-lying emitting states takes place. In the case of europium, the emitting state ($^5D_0$) is located at 579 nm from the ground state ($^7F_0$) while the main emission proceeds to a higher state of the ground state manifold ($^7F_2$), at about 615 nm. As is indicated by the formula above for the chloride, europium is ninefold coordinate, i.e., it can accommodate nine bonds, even though the europium ion is 3+.

### Sensitization with Organic Ligands

We previously[1] demonstrated the feasibility of tagging explosives traces via sensitized europium complexes, namely those involving ortho-phenanthroline (OP) and thenoyltrifluoroacetone (TTFA) as sensitizing ligands. These are bidentate ligands[1], such that four of them can bind to europium, leaving one potential binding site available. The binding to the explosive is to the ubiquitous $NO_2$ moiety. It is not clear to us at this time whether the binding to the explosive involves the ninth binding site or whether one of the sensitizing ligands is displaced on the binding to the explosive. We suspect the former, especially in the case of Eu-OP because OP is a strong ligand. The procedure described previously[1] involves spotting filter paper with the explosive and then spotting with the europium complex, or immersing the filter paper briefly in a (methanol) solution of the europium complex. At this stage, there is unreacted europium complex on the paper, precluding the visualization of the tagged explosive. A postreaction brief water rinse then quenches the luminescence of the unreacted complex, but not that of the explosive-bound complex. The explosive visualization is thus accomplished. The quenching of the unreacted Eu-OP is better than that of Eu-TTFA (steric hindrance inhibiting approach by water molecules?). The disadvantage of the Eu-OP complex is that it is best compatible with shortwave UV excitation. Laser excitation, needed for time-resolved visualization to suppress background fluorescence, is not easily accomplished in the deep UV, but is facile in the near UV. Today, there are commercial lasers with high powers, even in compact units that operate on ordinary household current or on car batteries (as in military vehicles). These lasers operate at 355 nm, a wavelength magnificently matched to the TTFA sensitizing ligand, as revealed by excitation spectroscopy. They are pulsed, operating at many kHz. Given the long europium luminescence lifetime, they thus act as if they were CW, and this makes facile time-resolved visualization possible via a simple mechanical light chopper and observation by the naked eye[8]. The principle is depicted in Fig. 3.

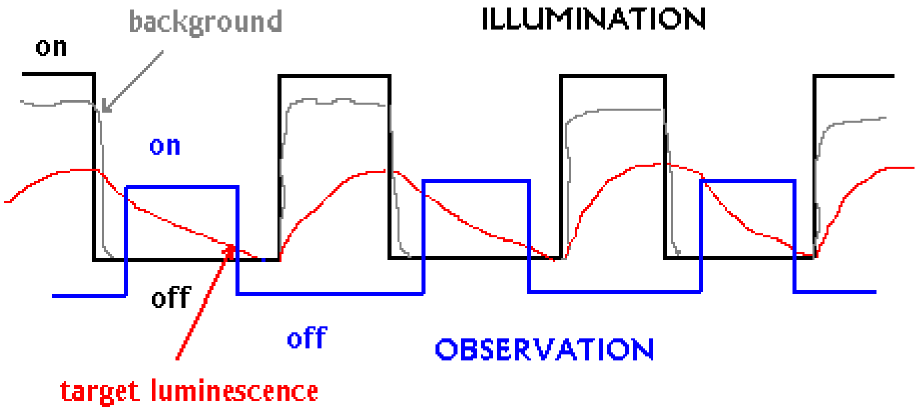

**FIGURE 3**. Principle of time-resolved luminescence visualization.

## *Results*

To take advantage of the chemical virtues of the OP ligand and the sensitizing advantage of the TTFA ligand, in order to remedy the sensitivity problem described in ref. [1], we prepared the mixed-ligand complex by simply mixing in methanol europium chloride, OP, and TTFA in 1:3:3 molar ratio. The excess ligand ensures the fullest possible europium coordination and also the presence in the complex of at least one OP ligand. Good water quenching of unreacted complex was achieved, as well as high luminescence intensity of explosive-bound complex under 355-nm excitation. In our earlier work[1], the UV excitation was at about 1 $mW/cm^2$, via a hand-held UV lamp. Such lamps have broad spectral output, much of which actually is not useful for europium luminescence sensitization. The sensitivity then was a factor of ten less than that of the usual ETK colorimetric sensitivity for Type II explosive. With 15-mW power at 355 nm (beam diameter at the explosive of 1 cm), using the laser and either direct visual observation or the time-resolved visualization apparatus of ref. [8], the luminescence sensitivity was a factor of ten better than the corresponding colorimetric one. No photobleaching was observed at that laser intensity level. Today, there are similar lasers on the market that deliver powers of well over 1 W. The time-resolved apparatus involves a simple mechanical light chopper with six openings, with the chopper wheel rotating at 50 Hz. This rotation rate is commensurate with the europium luminescence lifetime, and not so high as to be dangerous. Each chopper blade opening subtends an angle of 20 degrees. Thus, each intervening closing subtends an angle of 40 degrees. The laser is focused on the chopper blade and is beyond it dispersed to illuminate the sample. The visual observation, without any optics and also through the chopper blade, is done at 90 degrees from the laser (with respect to the blade's axis of rotation), such that when an opening is in front of the laser, a closing is in front of the eye. The closings are deliberately made considerably larger than the openings because otherwise our stereovision would severely restrict the field of view within which the time-resolved visualization is effective. The achieved sensitivity depends on the concentration of the europium complex. If it is too high, then there is insufficient luminescence quenching by water, and if it is too low, then there is insufficient intensity of the bound complex. We find that about 0.1 m*M* concentration is optimal.

## Nanocrystal Sensitization

It would be advantageous to be able to sensitize the emitting europium state directly, because then the very compact flashlight battery-operated laser used in our ordinary field prototype would be employable. This has until now not been possible, for quantum-mechanical reasons discussed previously[1]. However, with the advent of photoluminescent semiconductor nanocrystals, also referred to as quantum dots, nanoparticles, or nanocomposites, it occurred to us that the above direct sensitization might now become possible. Indeed, we demonstrated this direct sensitization, for the first time, we believe, for europium via CdS nanocrystals[1]. Unfortunately, these nanocrystals still require UV excitation. Thus, we explored the utilization of CdSe nancrystals, mostly referred to as quantum dots (Qdots). Qdots emitting in the orange, as needed for the requisite spectral overlap with the emitting europium state, have just become commercially available. However, the industry, still in its infancy, currently focuses on bioscience. Thus, the available Qdots, functionalized for biological purposes, are not designed for binding to europium. However, the donor-acceptor energy transfer is not actually dependent on chemical binding. It suffices that the donor and acceptor be in close proximity. The energy transfer efficiency depends on $R^{-6}$, where R is the distance between the donor and the acceptor. Of course, chemical binding is ideal because then small R is assured. Otherwise, one has to physically try to arrange things to produce a small R, which might not be trivial at distances on the order of 1–10 nm.

Europium complexes, namely Eu-TTFA (with stoichiometric ligand excess to ensure fullest coordination), were prepared in the usual manner in methanol solution, at a concentration of 3 m*M*. We note here that for the present purposes, the ligands are there only to exclude the luminescence-quenching water, rather than to serve as sensitizers, a function the Qdots are now supposed to serve. The complexes were spotted (one drop) on thin-layer chromatography (TLC) plates (silica gel without UV indicator). This substrate was chosen to inhibit water quenching of Eu luminescence, an issue in that the Qdots used by us come in water suspension. Silica is a well-known desiccant, binding water. TLC plates are not an optimal substrate for practical explosives detection in the field, which is yet some time in the future. Instead, the present concern is with energy transfer feasibility assessment. Qdot™ 585 Streptavidin Conjugate was obtained from Quantum Dot Corp. (www.qdots.com). This is a water suspension of the Qdots, and was used as received, without the accompanying buffer solution. It was spotted directly in one-drop quantity over the Eu spots on the TLC plate. Control Eu spots and Qdot spots alone were also applied as controls.

## *Results*

The Qdots respond to both UV and green excitation. Under UV excitation, the luminescence (control and other spots) was intense indeed, very much more intense than that of the control Eu spots, which, under UV excitation, are no luminescence slouches themselves. Under 532-nm green excitation, the Qdots continued to exhibit very intense emission, whereas the Eu control spots exhibited no luminescence at all, as to be expected. In the TLC spots, the Qdot luminescence peaked at 590 nm (slightly longer than the manufacturer-specified 585 nm, presumably because of aggregation), with full width at half maximum of 32.5 nm. In terms of donor-acceptor overlap at 579 nm, one is thus still at 70% of maximum donor luminescence intensity, quite good. However, at the Eu emission wavelength, about 615 nm, one is still at

30% of maximum Qdot luminescence, which represents a very large background emission intensity if one seeks Eu emission resulting from energy transfer. We thus anticipated having to employ time-resolved spectroscopy to suppress this background in order to dig out from underneath the anticipated much weaker Eu luminescence. Gratifyingly, it was not necessary to do so. Our luminescence spectroscopy employed a 0.3-m monochromator equipped with a grating blazed at 500 nm and a photomultiplier tube of extended S-20 spectral response. A gateable photon counter served for signal processing. Under the 532-nm excitation, a clearly defined shoulder at the right wavelength (about 615 nm) and of the right width (5–10 nm) was found in the overlayed spots but not in the Qdot control spot, with the Eu control spots not exhibiting any luminescence at all. This represented a Qdot-to-Eu energy transfer yielding Eu emission of intensity computed to be 4% of the maximum Qdot emission intensity. Subsequently carried out time-resolved spectroscopy, using the same instrumentation, and a mechanical light chopper to chop the excitation light, produced the characteristic peak at 615 nm without interference from the Qdot luminescence. The 615 nm Eu emission was 5% as intense as the 590-nm Qdot emission. The europium luminescence lifetime was also measured and yielded about 0.4 ms, in keeping with expectation[6]. Note here that there is a loss of Eu emission intensity that necessarily accompanies time-resolved spectroscopy because of the time delay between excitation source cut-off and gated measurement. Note also that the instrumentation is less sensitive at 615 nm than at 590 nm, and that the (raw) percentage figures reported above do not take this sensitivity difference into account. We construe our results as impressive energy transfer, considering that the above-discussed R proximity between Qdot and Eu was not achieved by chemical binding, but by merely placing drops over each other on a porous solid substrate. It should also be noted that the streptavidin conjugate is a very large entity (tens of kDa), such that close proximity between the sensitizing Qdot and the europium ion is inhibited. To put the spectroscopic results in perspective, we finally subjected the samples to the time-resolved visualization with the earlier-described apparatus, replacing the 355-nm laser with the flashlight battery operated green laser. Albeit weakly, the superposed Qdot/Eu complex spots were observable via the characteristic Eu emission, whereas the bare Eu-TTFA and the bare Qdot spots, were not.

## DISCUSSION

### ETK Approach

We emphasize that there is no chemistry involved in our fluorescence approach over the colorimetric ETK. We are simply taking advantage of the fact that the colorimetric products are also fluorescent, as discussed already[1], with the exception of Type IV explosive. As physicists, we defer here to the chemistry community regarding the explanation of how the chlorate or bromate function in a manner analogous to the nitro group in terms of the subsequent Griess reaction.

The experimental fluorescence investigation of the important issue of false positives would be very protracted and beyond the scope of the present account. However, the issue has no doubt been considered during the development of the commercial ETK, and the pertinent chemistry is long established (e.g., see ref. [5]). We note in this regard that a spurious compound that on ETK treatment would produce intense fluorescence would also produce intense color. Thus, we trust that this issue will not be severe, especially since field tests by rights should be considered presumptive only, to be followed by specific laboratory tests.

### Europium Approach

In the *in situ* time-resolved energy transfer-based detection of traces of explosives, our focus to date has been on the achievement of sensitivity and on the pertinent instrumentation. From an instrumental perspective, the implementation should be readily achievable in a field configuration. The Eu-OP/TTFA

strategy described is sufficiently good at this time to be practical as well. However, the issues of false positives and of global applicability still have to be assessed. Given that field tests are presumptive and that we believe that the ubiquitous $NO_2$ and analogous functionalities are being targeted, we do not foresee a major problem. The Qdot industry is as yet not considering the antiterrorist or forensic science market. Thus, it will still take some time to translate the reported sensitization feasibility demonstration to practicality. In contrast, the ETK chemistry-based photoluminescence field detection is mature and could be implemented in practice in short order.

The described energy transfer situation has implications beyond forensic science, namely in bioscience endeavors, such as studies of protein folding, for instance. It has not escaped our attention that the sensitive photoluminescence detection of traces of explosives suggests extension to the chemical and biological weapons of mass destruction arena, and we are conducting studies along those lines[10].

## ACKNOWLEDGMENTS

This material is based on work supported by the National Science Foundation and the Intelligence Technology Innovation Center through the joint “Approaches to Combat Terrorism” Program Solicitation NSF 03-569 (NSF 0345152). We are greatly indebted to Mr. David Turner of the Lubbock County Bomb Squad for making available to us numerous samples of explosives.
